# Delphinidin Reduces Cell Proliferation and Induces Apoptosis of Non-Small-Cell Lung Cancer Cells by Targeting EGFR/VEGFR2 Signaling Pathways

**DOI:** 10.1371/journal.pone.0077270

**Published:** 2013-10-04

**Authors:** Harish Chandra Pal, Samriti Sharma, Leah Ray Strickland, Jyoti Agarwal, Mohammad Athar, Craig A. Elmets, Farrukh Afaq

**Affiliations:** 1 Department of Dermatology, University of Alabama at Birmingham, Birmingham, Alabama, United States of America; 2 Comprehensive Cancer Center, University of Alabama at Birmingham, Birmingham, Alabama, United States of America; University of Kentucky, United States of America

## Abstract

Epidermal growth factor receptor (EGFR) and vascular endothelial growth factor receptor 2 (VEGFR2) have emerged as two effective clinical targets for non-small-cell lung cancer (NSCLC). In the present study, we found that delphinidin, an anthocyanidin, present in pigmented fruits and vegetables, is a potent inhibitor of both EGFR and VEGFR2 in NSCLC cells that overexpress EGFR/VEGFR2. Using these cells, we next determined the effects of delphinidin on cell growth and apoptosis *in vitro* and on tumor growth and angiogenesis *in vivo*. Delphinidin (5-60 µM) treatment of NSCLC cells inhibited the activation of PI3K, and phosphorylation of AKT and MAPKs. Additionally, treatment of NSCLC cells with delphinidin resulted in inhibition of cell growth without having significant toxic effects on normal human bronchial epithelial cells. Specifically, treatment of NCI-H441 and SK-MES-1 cells with delphindin (5-60 µM) resulted in (i) cleavage of PARP protein, (ii) activation of caspase-3 and -9, (iii) downregulation of anti-apoptotic proteins (Bcl2, Bcl-xL and Mcl-1), (iv) upregulation of pro-apoptotic proteins (Bax and Bak), and (v) decreased expression of PCNA and cyclin D1. Furthermore, in athymic nude mice subcutaneously implanted with human NSCLC cells, delphinidin treatment caused a (i) significant inhibition of tumor growth, (ii) decrease in the expression of markers for cell proliferation (Ki67 and PCNA) and angiogenesis (CD31 and VEGF), and (iii) induction of apoptosis, when compared with control mice. Based on these observations, we suggest that delphinidin, alone or as an adjuvant to current therapies, could be used for the management of NSCLC, especially those that overexpress EGFR and VEGFR2.

## Introduction

Lung cancer is a major health problem in the United States accounting for approximately 28% of all cancer-related deaths. Moreover, a total of 228,190 new cancer cases and 159,480 deaths from lung cancer have been projected to occur in the United States in 2013 [1]. The two major forms of lung cancer are small-cell lung cancer (SCLC) and non-small-cell lung cancer (NSCLC), which comprise approximately 15 and 85% of all cases respectively. Lung cancer has proven difficult to control with conventional therapeutic and surgical approaches leading to poor prognosis. Indicative of this poor prognosis, the overall 5-year survival rate is only 15% for NSCLC [2,3]. Clearly, investigation of alternative treatment options for NSCLC is warranted and will hopefully lead to alleviation of this major burden of mortality.

The epidermal growth factor receptor (EGFR/HER1/ErbB1) is a type 1 transmembrane receptor tyrosine kinase (RTK) of the ErbB family. It is composed of an extracellular-binding domain and an intracellular domain containing tyrosine kinase activity [4]. It is well established that EGFR is overexpressed in approximately 95% of all solid tumors [5,6]. Furthermore, it is known to be a critical oncogenic driver that arises in over 60% of NSCLC cases [7] and approximately 30% of breast cancers [8]. In addition, the vascular endothelial growth factor receptor 2 (VEGFR2) has been reported to be overexpressed in several tumors including lung cancer [9]. Moreover, the overexpression of both EGFR and VEGFR2 is associated with chemoresistance and is correlated with a poor prognosis [10,11]. Aberrant activation of EGFR and VEGFR2 triggers the phosphorylation of a multitude of proteins, including the PI3K/AKT and MAPK pathways which are involved in cell survival, apoptosis and angiogenesis [12,13]. Due to potential crosstalk and a well-established role in tumor growth and angiogenesis, inhibition of both EGFR and VEGFR2 signaling may improve the clinical outcome of advanced NSCLC patients. Different approaches have been adopted to simultaneously block EGFR and VEGFR2 signaling. Combinations of two specific inhibitors and single agents that target these receptors have been used; however their use in patients met with unacceptable toxicities.

There is an urgent need to develop multi-targeted therapeutic natural agent that blocks RTK activities and downstream signaling of both EGFR and VEGFR2 to improve therapeutic efficacy and minimize toxicity to normal host cells. One such agent is delphinidin, a dietary anthocyanidin known to be abundantly present in many pigmented fruits (pomegranates, berries and dark grapes) and vegetables (eggplants, tomatoes, carrots and red onions) [14]. It possesses anti-oxidant [15], anti-inflammatory [16,17], anti-proliferative [18] and anti-cancer activities [19]. In addition, delphinidin has been shown to inhibit the EGFR signaling pathway as well as invasion of breast cancer cells [20]. However, most of the anti-cancer and anti-angiogenic activities reported in the literature are based on *in vitro* studies. The goal of the present study was to determine the effects of delphinidin on cell growth and apoptosis *in vitro* and on tumor growth and angiogenesis *in vivo* in NSCLC cells. Our results indicated that delphinidin is a potent inhibitor of both EGFR and VEGFR2 in NSCLC cells. In addition, delphindin inhibited the activation of PI3K, and phosphorylation of AKT and MAPKs. This resulted in cell growth inhibition and induction of apoptosis in NSCLC cells. Furthermore, delphindin treatment inhibited the growth of NSCLC xenografts in nude mice which was associated with a decrease in the expression of markers for cell proliferation and angiogenesis as well as an induction of apoptosis.

## Materials and Methods

### Materials

Delphinidin (>98% pure) was purchased from Extrasynthase (Lyon, France). The monoclonal and polyclonal antibodies for EGFR and phospho-EGFR, VEGFR2 and phospho-VEGFR2, ERK1/2 (phospho-p44/42, Thr^202^/Tyr^204^), JNK1/2 (phospo-p54/46, Thr^183^/Tyr^185^), p38 (phospho-p38, Thr^180^/Tyr^204^), PI3K, phopho AKT, Bcl2, Bcl-xL, Mcl-1, Bax, Bak, cyclin D1, PARP, caspase-3 and -9 were obtained from Cell Signaling Technology (Beverly, MA). Polyclonal antibodies for VEGF, PCNA and Ki67 were purchased from Santa Cruz Biotechnology, Inc. (Santa Cruz, CA). Anti-mouse CD31 antibody was obtained from BD Biosciences (San Jose CA). Anti-mouse or anti-rabbit secondary horseradish peroxidase conjugate was obtained from Millipore Corporation (Billerica, MA).

### Treatment of cells

Human NSCLC cells NCI-H441, SK-MES-1 and A549 were obtained from American Type Culture Collection (Manassas, VA). NCI-H441 cells were cultured in RPMI1640 medium (HyClone Laboratories Inc., Logan, UT), SK-MES-1 cells were cultured in EMEM medium (HyClone Laboratories Inc., Logan, UT), and A549 cells were cultured in Ham’s F-12K medium (Mediatech Inc., Manassas, VA) supplemented with 10% heat-inactivated fetal bovine serum and 100 mg/ml penicillin-streptomycin. Normal human bronchial epithelial (NHBE) cells were obtained from Clonetics Airways Epithelial Cell Systems (Cambrex Bio Science, Walkersville Inc., MD) and cultured in Bronchial Epithelial Growth Media supplemented with growth factors (Cambrex Bio Science, Walkersville Inc., MD). The cells were maintained under standard cell culture conditions at 37°C and 5% CO_2_ in a humid environment. Delphinidin (dissolved in DMSO) was used for the treatment of cells. The final concentration of DMSO used was 0.1% (v/v) for each treatment. For dose-dependent studies NCl-H441 and SK-MES-1 cells were treated with delphinidin (5-60 µM) for 3 and 48 hrs in complete cell medium. Control cells were treated with the vehicle alone. In additional experiments, serum starved NCl-H441 and SK-MES-1 cells were treated with delphinidin (5-60 µM) for 3 hrs and then incubated without or with EGF (50 ng/ml; 15 min) or without and with VEGF (20 ng/ml; 30 min).

### Preparation of cell lysates

After cell treatment with delphinidin, the medium was aspirated and the cells were washed with PBS (10 mmol/l, pH 7.45). The cells were then incubated in an ice cold lysis buffer (10 mM HEPES (pH 7.9), 100 mM KCl, 10 mM EDTA, 20 mM EGTA, 100 mM DTT, 20 mM PMSF, 0.5% NP-40 with freshly added protease inhibitors leupeptin, aprotinin and benzamidine) for 20 min. The cells were harvested and the lysate was collected in a microfuge tube and passed through a 21.5-G needle to break up the cell aggregates. The lysate was cleared by centrifugation at 14,000g for 10 min at 4°C, and the supernatant (total cell lysate) collected, aliquoted and then used on the day of preparation or immediately stored at -80°C for use at a later time.

### Western blot analysis

For western blotting, 30-50 µg protein was resolved over 8-12% Tris-glycine gels and transferred to a nitrocellulose membrane. Briefly, the membrane was blocked and probed with appropriate primary and secondary antibody HRP conjugate followed by chemiluminescence and autoradiography as described earlier [20].

### Cell viability assay

The effect of delphinidin on cell viability was determined by 3-[4,5-dimethylthiazol-2-yl]-2,5-diphenyl tetrazolium bromide (MTT) assay. Cells (NHBE, NCI-H441, A549, and SK-MES-1) were plated in a 96-well microtiter plate and treated with 5-100 µM concentrations of delphinidin for 48 hrs. 1/10 volume of 10xMTT solution (5 mg/ml in PBS) was added to each well and incubated for 2 hrs and absorbance was recorded on a microplate reader at 540 nm after solubilizing reduced MTT with DMSO. The effect of delphinidin on growth inhibition was assessed as percent cell viability where DMSO-treated cells were taken as 100% viable.

### Treatment of athymic nude mice

Four-five weeks old female athymic (nu/nu) nude mice were purchased from NCI-Frederick National Laboratory for Cancer Research and housed under pathogen-free conditions with a 12 hrs light/12 hrs dark schedule in the Animal Resource Facility at the University of Alabama at Birmingham in accordance with the Institutional Animal Care and Use Committee guidelines. The animal protocol used in this study was approved by the Institutional Animal Care and Use Committee (IACUC) of the University of Alabama at Birmingham, and the animal protocol number is 120609365. Animals were fed with phytochemical free diet AIN-76 SEMI PD (Test Diet, Richmond, IN) *ad libitum*. The mice were subcutaneously injected with a fixed number of NCI-H441 cells (4x10^6^ in 50 µl RPMI + 50 µl matrigel) in each flank to initiate tumor growth. Animals were then randomly divided into three groups with 6 animals in each group. The first group of animals received an i.p. injection of DMSO (100 µl) and served as a control. The animals of group 2 and 3 received an i.p. injection of delphinidin (1 mg/animal and 2 mg/animal respectively) in 100 µl of DMSO three times/week. Tumor sizes were measured twice a week, and tumor volume was calculated by the formula ½ (*L*_1_ × *L*_2_ × *H*), where *L*_1_ is the long diameter, *L*_2_ is the short diameter, and H is the height of the tumor. All animals were sacrificed by CO_2_ inhalation and death was confirmed by cervical dislocation when tumors reached a volume of approximately 1200 mm^3^ in the control group. Similar experiments were then performed with SK-MES-1 cells. All procedures conducted were in accordance with the guidelines for the use and care of laboratory animals and approved by IACUC.

### Immunohistochemistry and immunofluorescence staining

Five micrometer sections were cut, deparaffinized in xylenes, rehydrated in ethanol, and washed in phosphate-buffered saline. For antigen retrieval, sections were heated at 95°C for 30 min in citrate buffer (pH 6.0). Briefly, sections were then incubated with primary antibodies overnight at 4°C, followed by incubation with a specific horseradish peroxidase-labeled secondary antibody for 1 hr at room temperature. Next, the sections were incubated with diaminobenzidene peroxidase substrate solution for 2 min and counterstained with Mayer’s Hematoxylin solution. For immunofluorescence staining after antigen retrieval, sections were incubated with primary antibodies overnight at 4°C, followed by incubation with Alexafluor-488 labeled secondary antibody for 1 hr at room temperature. Slides were mounted with Vectashield mounting media containing DAPI and were analyzed under fluorescence microscope.

### Statistical analysis

The results are expressed as the mean ± SEM. Statistical analysis of all the data was performed by Student’s t-test. The *p* value <0.05 was considered statistically significant.

## Results

### Delphinidin treatment inhibits the constitutive and EGF-induced phosphorylation of EGFR in NCl-H441 cells

**Figure 1:**
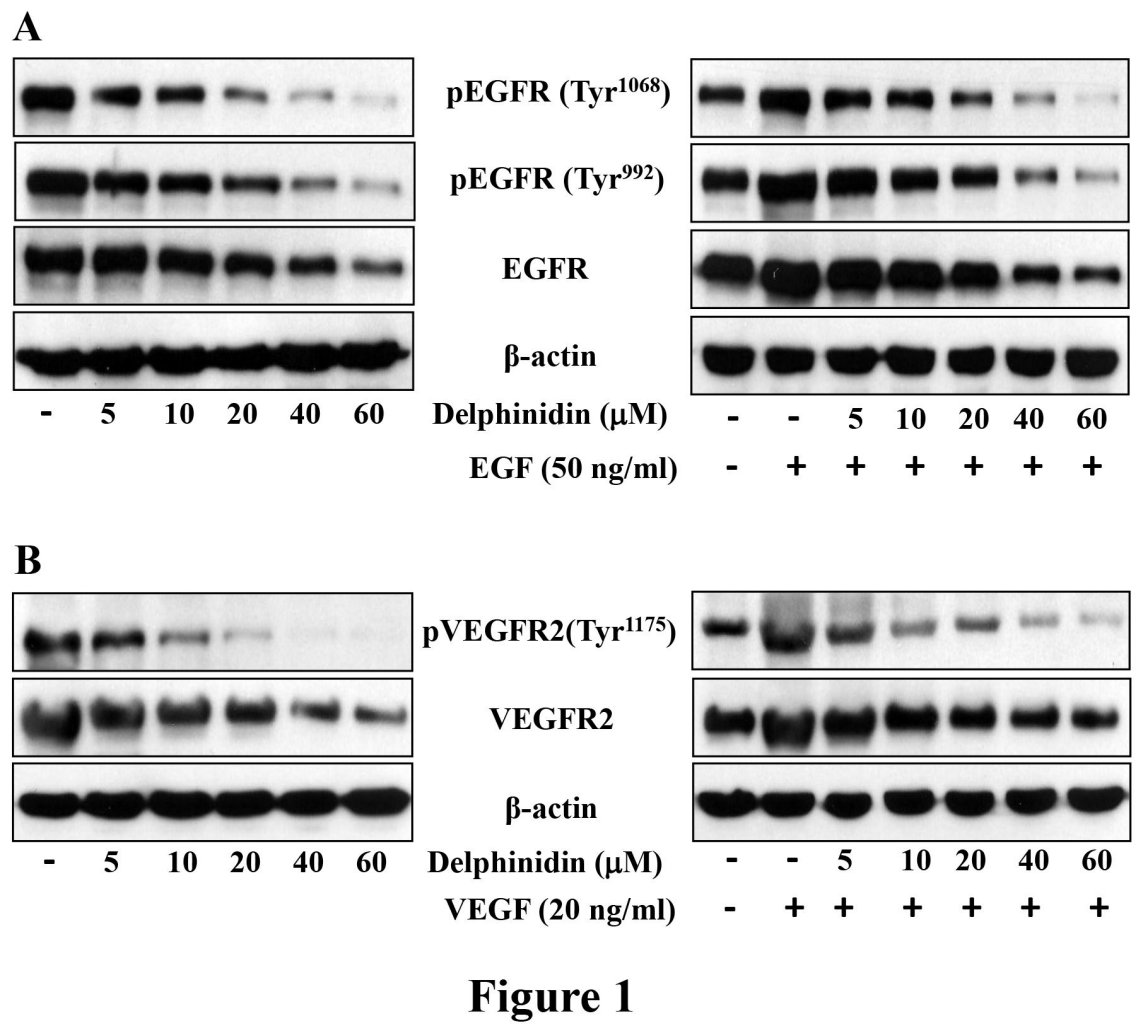
Effect of delphinidin treatment on the constitutive and EGF- and VEGF-induced phosphorylation of EGFR and VEGFR2 in NCl-H441 cells. [A] NCl-H441 cells were treated with delphinidin (5-60 µM) for 3 hrs in complete cell medium (left panel). In the right panel, serum starved NCl-H441 cells were treated with delphinidin (5-60 µM) for 3 hrs and then incubated without or with EGF (50 ng/ml) for 15 min. After treatment cells were harvested and cell lysates were prepared and the expression EGFR and phosphorylated-EGFR was determined. [B] NCl-H441 cells were treated with delphinidin (5-60 µM) for 3 hrs in complete cell medium (left panel). In the right panel, serum starved NCl-H441 cells were treated with delphinidin (5-60 µM) for 3 hrs and then incubated without or with VEGF (20 ng/ml) for 30 min. After treatment cells were harvested and cell lysates were prepared and the expression VEGFR2 and phosphorylated VEGFR2 was determined. Equal loading of protein was confirmed by stripping the immunoblot and reprobing it for β-actin. The immunoblots shown here are from a representative experiment repeated three times with similar results.

Aberrant activation and overexpression of EGFR leads to dysregulation of signaling pathways crucial for cell proliferation, survival, cancer progression, angiogenesis and metastatis [21]. EGFR overexpressing NCI-H441 cells were treated with delphinidin (5-60 µM; 3 hrs) to determine its effect on EGFR expression. As shown in Figure 1A, delphinidin treatment significantly reduced expression and phosphorylation of EGFR in NCI-H441 cells. In addition, overexpression of its ligand EGF, which activates EGFR, plays an important role in tumorigenesis of NSCLC [22]. We therefore determined the effect of delphinidin on EGF-induced phosphorylation of EGFR and found that its treatment significantly inhibited EGF-induced activation and phosphorylation of EGFR (Figure 1A). A similar effect of delphinidin on the phosphorylation of EGFR was also observed in SK-MES-1 cells (data not shown). This demonstrates that delphinidin treatment reduces both constitutive and EGF-induced phosphorylation of EGFR.

### Delphinidin treatment inhibits the constitutive and VEGF-induced phosphorylation of VEGFR2 in NCl-H441 cells

Similar to EGFR, overexpression and aberrant activation of VEGFR2 has also been reported in several cancer cells, including NSCLC [23]. Like EGFR, VEGFR2 undergoes dimerization and VEGFR ligand-dependent phosphorylation and activation resulting in dysregulation of downstream signaling. This then triggers mitogenic, chemotactic, and pro-survival signals, along with stimulation of tumor vessel formation [24]. Therefore, we also evaluated the effect of delphinidin (5-60 µM; 3 hrs) on expression of VEGFR2 in NCH-H441 cells and found that its treatment significantly reduced the expression of VEGFR2 in NCI-H441 cells (Figure 1B). In addition, delphinidin treatment inhibited VEGF- induced activation and phosphorylation of VEGFR2 (Figure 1B). A similar effect of delphinidin on the phosphorylation of VEGFR2 was also observed in SK-MES-1 cells (data not shown). These results clearly demonstrate that delphinidin treatment inhibits constitutive and VEGF-induced phosphorylation of VEGFR2.

### Delphinidin treatment inhibits the protein expression of PI3K and phosphorylation of AKT and MAPKs in NCl-H441 and SK-MES-1 cells

**Figure 2:**
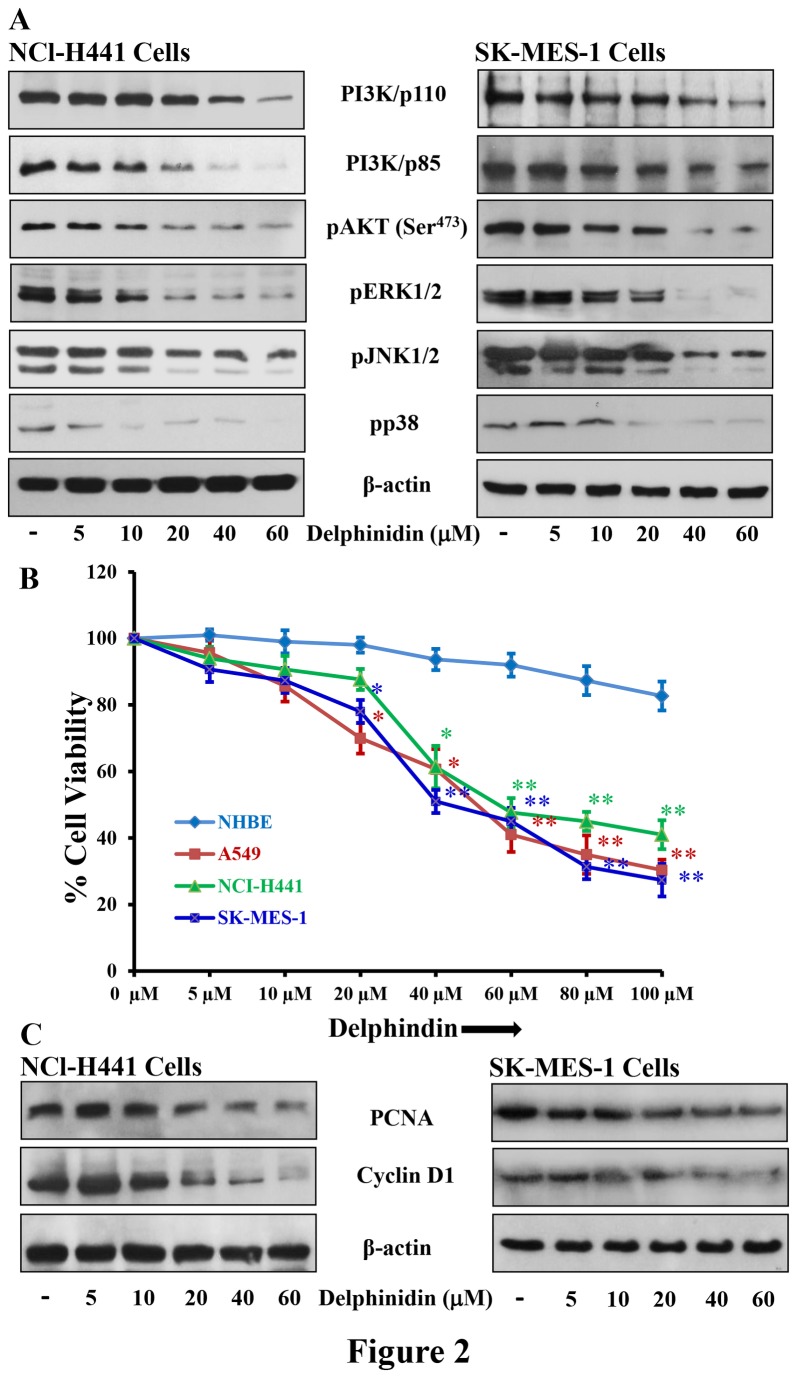
Effect of delphinidin treatment on cell viability protein expression of PI3K, phosphorylation of AKT, MAPKs and expression of PCNA and cyclinD1 in NSCLC cells. [A] NCI-H441 and SK-MES-1 cells were treated with 5-60 µM delphindin for 48 hrs to determine its effect on protein expression of PI3K, phosphorylation of AKT and MAPKs. After treatment cells were harvested, cell lysates were prepared and protein was subjected to SDS-PAGE followed by immunoblot analysis and chemiluminescence detection. Equal loading of protein was confirmed by stripping the immunoblot and reprobing it for β-actin. The immunoblots shown here are from a representative experiment repeated three times with similar results. [B] Cell viability of A549, NCI-H441, SK-MES-1 and NHBE cells treated with 5-100 µM of delphinidin for 48 hrs, was determined by MTT assay as described in “Materials and Methods”. Data shown are mean ± SEM of three separate experiments in which each treatment was repeated in 10 wells. [C] NCI-H441 and SK-MES-1 cells were treated with 5-60 µM delphindin for 48 hrs to determine its effect on protein expression of PCNA and cyclinD1. After treatment cells were harvested, cell lysates were prepared and protein was subjected to SDS-PAGE followed by immunoblot analysis and chemiluminescence detection. Equal loading of protein was confirmed by stripping the immunoblot and reprobing it for β-actin. The immunoblots shown here are from a representative experiment repeated three times with similar results.

Resistance to EGFR inhibitors is associated with activation of PI3K/AKT and MAPKs signaling pathways [25]. EGFR/PI3K/AKT/MAPKs signaling plays a pivotal role in the tumorigenesis, enhanced cell proliferation, angiogenesis, and inhibition of apoptosis in various human malignancies, including NSCLC [21,26]. Once PI3K/AKT signaling is activated by EGFR, it phosphorylates multiple downstream targets involved in key cellular processes including cell proliferation and apoptosis. Treatment of delphinidin (5-60 µM; 48 hrs) resulted in decreased expression of p85 (a regulatory subunit) and p110α (a catalytic subunit) of PI3K in NCI-H441 and SK-MES-1 cells (Figure 2A). PI3K regulates phosphorylation and activation of AKT, which promotes cell survival by blocking the functions of pro-apoptotic proteins and promoting the induction of cell survival proteins [27]. As a consequence of PI3K inhibition by delphinidin, AKT phosphorylation was significantly reduced in both NCI-H441 and SK-MES-1 cells (Figure 2A).

The MAPKs pathway is another signaling cascade that is activated by EGFR and plays an important role in the regulation of many cellular responses including cell proliferation and apoptosis [28]. The MAPKs family is comprised of three major subgroups: ERK (extracellular signal-regulated protein kinase), JNK (c-Jun N-terminal kinase) and p38 MAPK [29]. ERKs are mainly involved in regulation of mitogen-activated proliferation/differentiation factors, whereas JNK and p38 MAPKs perform functions related to apoptotic cell death [30]. Therefore, we also evaluated the effect of delphinidin on MAPKs signaling in NSCLC cells. We found that delphinidin treatment significantly reduced phosphorylation of ERK1/2, JNK1/2 and p38 in both NCI-H441 and SK-MES-1 cells (Figure 2A).

### Delphinidin treatment inhibits the growth of NSCLC cells

Since overexpression and aberrant activation of EGFR and its downstream signaling pathways is inhibited by delphinidin, we next examined its effect on the cellular proliferation of NSCLC cells by employing an MTT assay. NSCLC A549, NCI-H441 and SK-MES-1 cells overexpressing EGFR and VEGFR2 were treated with delphinidin (5-100 µM; 48 hrs). Results of the MTT assay showed that delphinidin was effective at significantly inhibiting cell growth of A549 (4-70%; p<0.05-0.01), NCI-H441 (6-59%; p<0.05-0.01) and SK-MES-1 cells (9-73%; p<0.05-0.01) (Figure 2B). The IC_50_ values of delphinidin were estimated to be 55, 58 and 44 µM for A549, NCI-H441 and SK-MES-1 cells respectively. We also examined the effects of delphinidin on the growth of normal human bronchial epithelial (NHBE) cells under similar conditions and found that these doses have only marginal effect on growth of these cells (Figure 2B).

### Delphinidin treatment inhibits the protein expression of cyclin D1 and PCNA in NCl-H441 and SK-MES-1 cells

The signaling pathways involved in activation of MAPKs also activate several nuclear proteins, which play a crucial role in cell cycle progression from G1 to S phase. Specifically, cyclin D1 has been identified as a key downstream effector of EGFR signaling in resistant NSCLC cells. Moreover, EGFR mutant NSCLC cells have higher expression of cyclin D1 [31]. Treatment of NCI-H441 and SK-MES-1 NSCLC cells with delphinidin (5-60 µM; 48 hrs) resulted in a significant reduction of cyclin D1 protein expression in a dose-dependent manner (Figure 2C). PCNA is a known marker of cellular proliferation active during the S and G2 phases of the cell cycle and plays an important role in the initiation of cell proliferation [32]. The increased expression of PCNA in cancer patients has been associated with a poor survival rate. In addition, EGFR also functions as a transcription factor and enhances cell proliferation by activating and stabilizing PCNA [33]. We therefore examined the effect of delphindin on PCNA expression and found that it significantly reduced PCNA protein expression in both NCI-H441 and SK-MES-1 cells (Figure 2C).

### Delphinidin treatment modulates the Bcl2 family protein expression, and induces cleavage of caspases and PARP in NCl-H441 and SK-MES-1 cells

**Figure 3:**
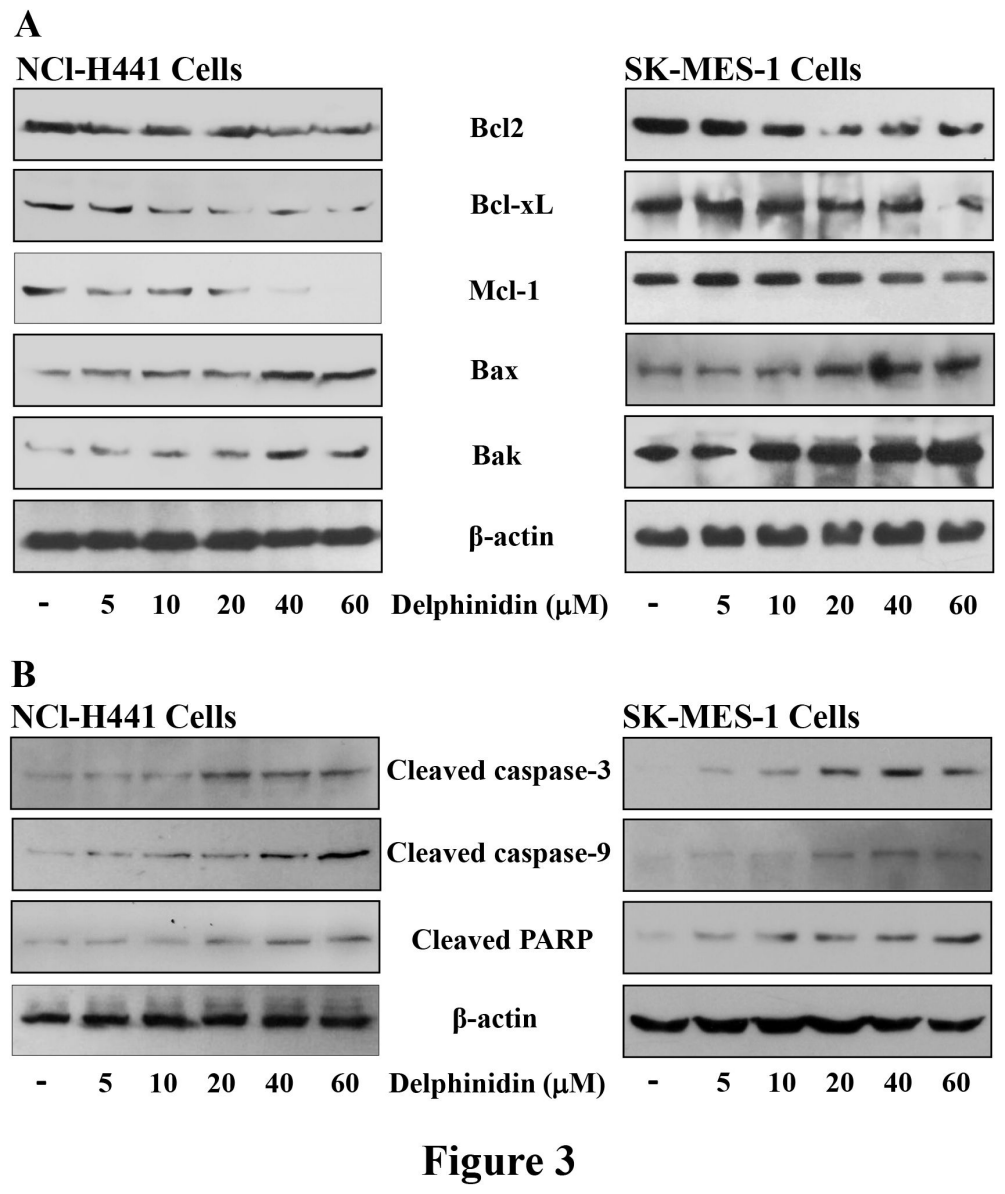
Effect of delphinidin treatment modulation of Bcl2 family proteins and cleavage of caspases and PARP in NSCLC cells. [A & B] NCI-H441 and SK-MES-1 cells were treated with 5-60 µM delphindin for 48 hrs to determine its effect on expression of Bcl2 family proteins and cleavage of caspases and PARP. After treatment cells were harvested, cell lysates were prepared and protein was subjected to SDS-PAGE followed by immunoblot analysis and chemiluminescence detection. Equal loading of protein was confirmed by stripping the immunoblot and reprobing it for β-actin. The immunoblots shown here are from a representative experiment repeated three times with similar results.

Activation of PI3K/AKT and MAPKs signaling by EGFR results in enhanced cell proliferation and angiogenesis, as well as inhibition of apoptosis. Treatment of EGFR overexpressing NSCLC cells with delphinidin resulted in a significant inhibition of PI3K/AKT and MAPKs signaling pathways. We next examined the effects of delphinidin on Bcl2 family proteins downstream of PI3K/AKT. Treatment of NCI-H441 and SK-MES-1 cells with delphinidin greatly reduced the expression of anti-apoptotic proteins such as Bcl2, Bcl-xL and Mcl-1 (Figure 3A). In addition, we found that the expression of pro-apoptotic proteins Bak and Bax was significantly increased in delphinidin treated NCI-H441 and SK-MES-1 cells (Figure 3A). Apoptosis is a highly regulated process that involves a cascade of events, including proteolytic activation of caspases. Within the apoptotic process caspases can act either as the initiators or executioners. Executioner caspases are activated from their pro-enzymatic form by the action of other caspases within a cascade reaction. Once activated, caspases cleave a variety of intracellular proteins, such as PARP and different protein kinases [34]. Therefore, we next examined whether delphinidin treatment (5-60 µM; 48 hrs) caused activation of caspases or cleavage of PARP, both of which are hallmarks of apoptosis. We found that delphinidin treatment resulted in the activation of caspase-9, caspase-3 and the consequent cleavage of PARP in both NCI-H441 and SK-MES-1cells (Figure 3B).

### Delphinidin treatment inhibits tumorigenicity of human NSCLC cells implanted in athymic nude mice

**Figure 4:**
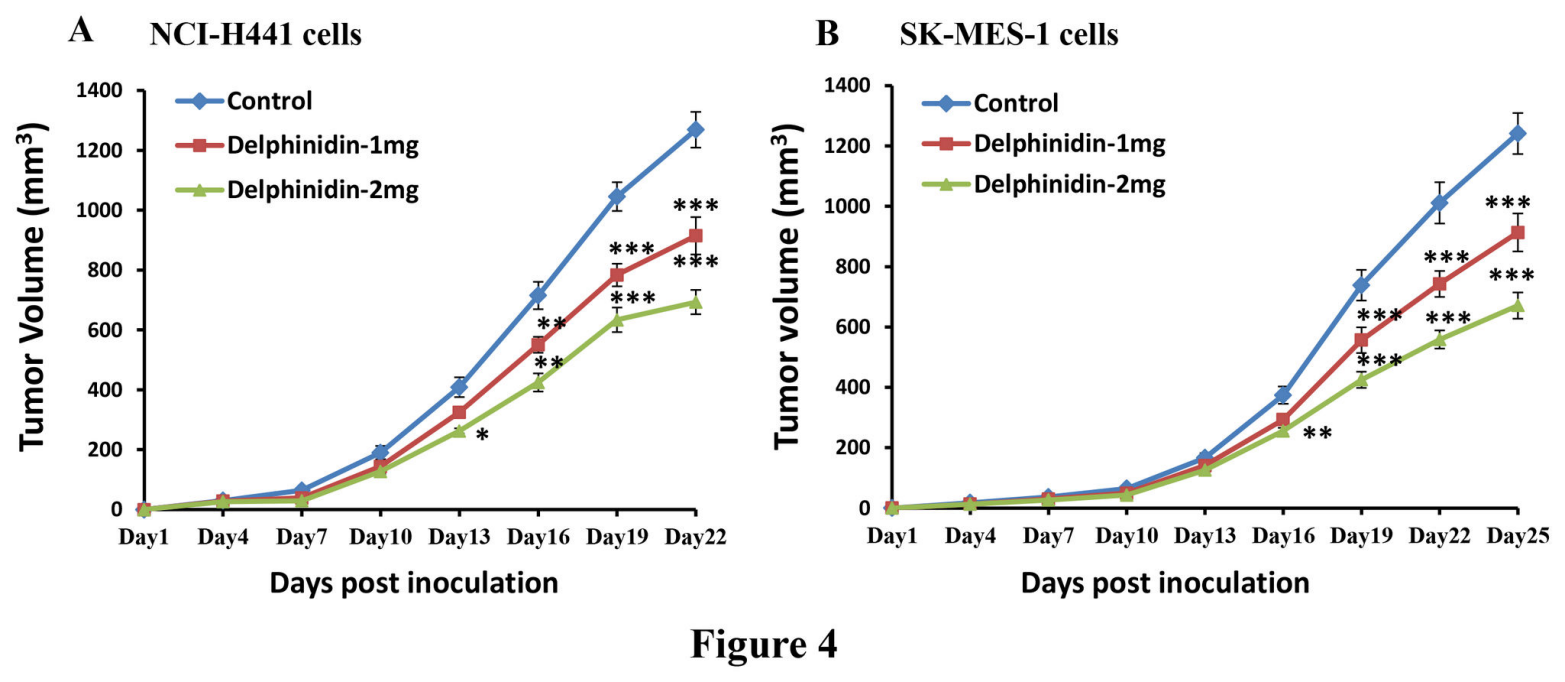
Effect of delphinidin treatment on human NSCLC tumor growth in athymic nude mice. [A] Eighteen athymic (*nu/nu*) female nude mice were subcutaneously injected with 4x10^6^ NCI-H441 cells (in 50 µl RPMI + 50 µl Matrigel) in each flank of mouse to initiate tumor growth and then randomly divided into three groups (six mice in each). Twenty-four hours after cell implantation, mice of the first group received i.p. injection of DMSO (100 µl) and served as control. The mice of group 2 and 3 received i.p. injection of delphinidin 1 mg/animal and 2 mg/animal respectively in 100 µl of DMSO three times/week. [B] Similar experiments were performed with SK-MES-1 cells. Once tumors started to grow, their sizes were measured and tumor volume was calculated by the formula ½(*L*_1_ × *L*_2_ × *H*), where *L*_1_ is the long diameter, *L*_2_ is the short diameter, and *H* is the height of the tumor. All animals were sacrificed when tumors reached a volume of 1200 mm^3^ in the control group. Average tumor volumes of the control and delphindin treated groups were plotted over days after tumor cell inoculation. Values represent mean ± SEM. *p<0.05, **p<0.01, and ***p<0.001 versus control group.

Since delphinidin effectively reduced cell growth and induced apoptosis of NSCLC cells *in vitro*, we next determined the effects of delphinidin on an *in vivo* xenograft mouse model implanted with NCI-H441 or SK-MES-1cells. In athymic nude mice implanted with EGFR and VEGFR2 overexpressing NSCLC cells, delphinidin treatment resulted in significant inhibition of tumor growth. Delphinidin treated mice implanted with NCI-H441 cells showed significant tumor growth inhibition (p<0.05-0.001) by 27.92 and 45.37% at a dose of 1 and 2 mg/animal respectively compared to the untreated control group (Figure 4A). The average tumor volume of the control group was 1268.62 mm^3^, whereas in the delphindin treated groups the average tumor volumes were 914.38 mm^3^ and 693.01 mm^3^ in mice that received 1 and 2 mg/animal delphinidin respectively (Figure 4A). In addition, delphinidin treatment also resulted in significant tumor growth inhibition in athymic nude mice implanted with SK-MES-1 cells. Treatment of delphinidin (1 and 2 mg/animal) resulted in 26.34 and 46.01% tumor growth inhibition as compared with control mice. These findings were statistically significant (p<0.05-0.001). Average tumor volume in delphinidin treated groups was significantly reduced (913.42 mm^3^ and 671.18 mm^3^) when compared with the untreated control group (1241.18 mm^3^) (Figure 4B).

### Delphinidin treatment inhibits markers of proliferation and induces apoptosis in tumors of athymic nude mice implanted with NSCLC cells

**Figure 5:**
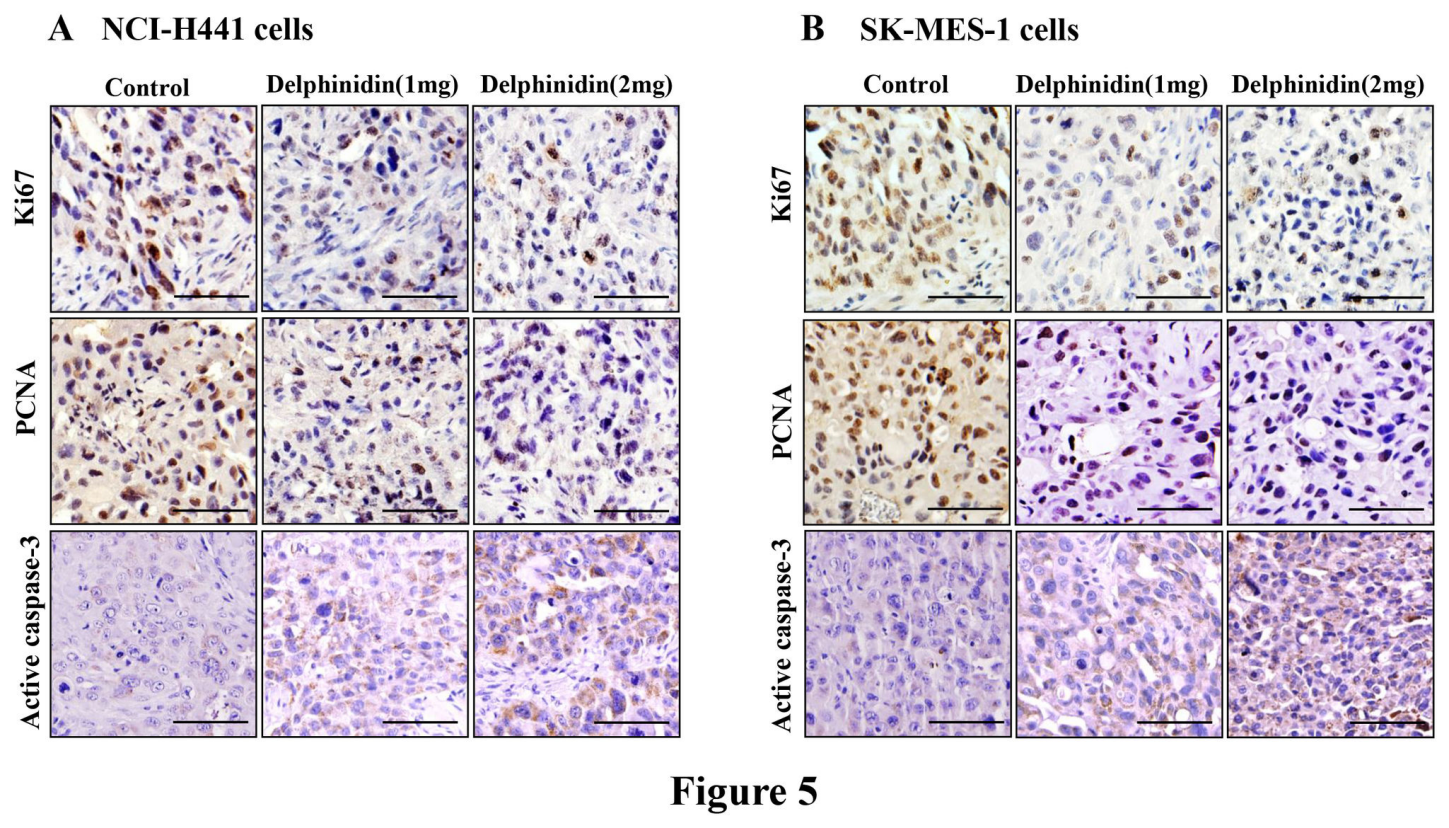
Effect of delphinidin treatment on markers of proliferation and apoptosis in tumors of athymic nude mice implanted with NSCLC cells. Athymic nude mice were implanted with [A] NCI-H441, and [B] SK-MES-1 cells. Mice were treated with delphinidin, tumor tissues were collected in 10% formalin and blocks were prepared in paraffin and immunostaining of Ki67, PCNA and cleaved caspase-3 were performed as described in “Materials and Methods”. Photomicrographs show representative pictures from three independent tumor samples. Bar = 20 µm.

Next, we examined the expression of molecules associated with cell proliferation and apoptosis in tumor xenografts by employing immunohistochemistry. As shown in Figure 5, results of histochemical analysis of tumor sections demonstrated that there was a significant decrease in the numbers and intensity of cell proliferation markers such as Ki67 and PCNA in delphinidin treated tumors as compared to their respective untreated control groups. In addition, when these tumors were evaluated for staining of active caspase-3, a hallmark of apoptosis, we found that there was a significant increase in the number and intensity of active caspase-3 staining in tumors of delphinidin treated mice (Figure 5A and 5B).

### Delphinidin treatment inhibits markers of angiogenesis in tumors of athymic nude mice implanted with NSCLC cells

**Figure 6:**
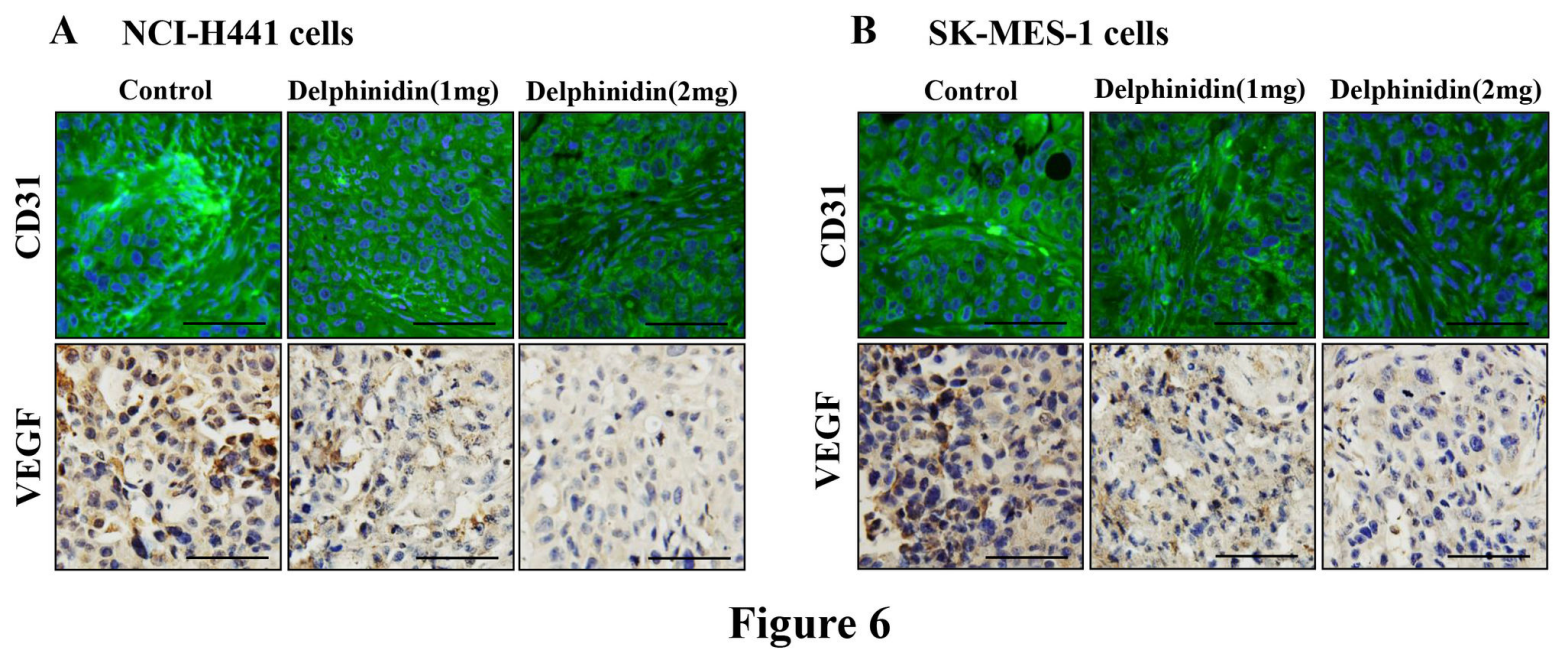
Effect of delphinidin treatment on markers of angiogenesis in tumors of athymic nude mice implanted with NSCLC cells. Athymic nude mice were implanted with [A] NCI-H441, and [B] SK-MES-1 cells. Mice were treated with delphinidin, tumor tissues were collected in 10% formalin and blocks were prepared in paraffin and immunofluorescence of CD31 and immunohistochemistry of VEGF were performed as described in “Materials and Methods”. Photomicrographs show representative pictures from three independent tumor samples. Bar = 20 µm.

Solid tumors recruit new blood vessels for growth, maintenance, and metastasis. Therefore, the use of agents that suppress tumor-induced development of new blood vessels are considered an important strategy for cancer treatment. Therefore many current clinical therapies target vascular endothelial growth factor (VEGF) and CD31. Tumor sections stained with anti-VEGF and anti-CD31 antibodies showed reduced intensity of staining in the delphinidin treated groups. This study clearly demonstrated that delphinidin treatment significantly reduced expression of VEGF and CD31 as compared to tumor sections of control mice (Figure 6A and 6B).

## Discussion

There has been great strides in unraveling the mysteries of cancer genetics and biology; however the most important task is to translate these discoveries into novel therapeutics that will improve patient outcomes. Targeted therapies are the future of cancer treatment and development of novel therapeutic inhibitors of signal transduction molecules, in particular RTK, is the focus of extensive research. Overexpression and aberrant activation of RTK such as EGFR and VEGFR2 are associated with higher proliferation rates, reduced apoptosis, increased angiogenesis and metastasis [35]. They thus present an attractive target for drug development against NSCLC [36,37]. Targeted therapies against EGFR andVEGFR2 are now widely used to treat NSCLC patients [38-41]. However, intrinsic resistances and unacceptable cytotoxic effects on normal cells largely limit the clinical application of these inhibitors. New strategies and novel agents are required to replace or complement current therapies. In the present study, we investigated the effect of delphinidin treatment on NSCLC cells that overexpress EGFR and VEGFR2.

We found that treatment of EGFR overexpressing NSCLC cells with delphinidin, reduced phosphorylation and expression of EGFR. The inhibitory effect of delphinidin was maintained even when these cells were challenged with their ligand, EGF, indicating that delphinidin strongly inhibited EGFR signaling in NSCLC cells (Figure 1A). In addition, VEGFR2 is another clinically validated therapeutic target in NSCLC [23,42]. Moreover, EGFR is known to regulate the production of VEGF, a pro-angiogenic factor [43]. The VEGF signaling pathway is critical for endothelial cell proliferation, migration, cell survival and for the induction of vascular permeability. Increased VEGF expression has been associated with resistance to EGFR inhibition in a human tumor xenograft model of NSCLC [44]. In the present study, delphinidin reduced the phosphorylation and expression of VEGFR2 in VEGF challenged VEGFR2 over expressing NSCLC cells (Figure 1B). We found that simultaneous inhibition of EGFR and VEGFR2 signaling pathways with delphinidin may offer greater anti-tumor efficacy for advanced NSCLC than inhibitors of either pathway alone. The other major advantage of delphinidin is that it is non-toxic to normal cells (Figure 2B).

Since, EGFR can interact with and activate PI3K/AKT and its downstream signaling components [45,46], sustained PI3K/AKT signaling activation has been implicated in the resistance of RTK inhibitors targeting EGFR signaling [47,48]. In the present study, delphinidin showed great potential to reduce the cell viability of different NSCLC cells (Figure 2B), also its treatment significantly reduced the phosphorylation of the PI3K/AKT signaling pathways, which are necessary for cell proliferation and survival (Figure 2A). In addition, activation of EGFR signaling has been implicated in a number of protein kinases including MAPKs associated with cell survival [49]. Delphinidin treatment of EGFR overexpressing NSCLC cells also significantly reduced phosphorylation of MAPKs such as ERK1/2, JNK1/2 and p38 (Figure 2A). These MAPKs phosphorylate a number of cellular substrates and thus play an important role in mediating cell proliferation and survival [50]. In addition, delphinidin treatment reduced the expression of PCNA and cyclin D1 (Figure 2C), proteins required for cell proliferation and cell cycle progression from G1 to S phase. Activation of EGFR, PI3K/AKT, or MAPK leads to down regulation of apoptosis; therefore, induction of apoptosis is one of the most obvious targets for cancer treatment [51,52]. Bcl2 family proteins are key regulators of apoptosis and include both anti-apoptotic proteins (Bcl2, Bcl-xL and Mcl-1) and pro-apoptotic proteins (Bax and Bak). A slight change in the balance of these proteins results either in inhibition or promotion of cell death. In the present investigation, delphinidin treatment of NSCLC cells resulted in decreased expression of anti-apoptotic proteins Bcl2, Bcl-xL and Mcl-1, whereas expression of pro-apoptotic proteins Bax and Bak was increased after delphinidin treatment (Figure 3A). Activation of caspases is an important event in the signal transduction of apoptosis. Once activated, caspases are responsible for the proteolytic cleavage of a broad spectrum of cellular targets ultimately leading to cell death. Known cellular substrates for caspases include major structural elements of the cytoplasm and nucleus, along with components of DNA repair machinery like PARP and protein kinases [34]. In the present investigation, delphinidin treatment resulted in significant activation of caspase-3 and -9 with concomitant PARP cleavage in NSCLC cells (Figure 3B).

To establish the relevance of these *in vitro* findings, we implanted athymic nude mice with human NSCLC (NCI-H441 or SK-MES-1) cells. Intraperitoneal administration of delphindin significantly slowed the progression of NCI-H441 and SK-MES-1 tumor growth in athymic nude mice (Figure 4A and 4B). We also found that there was a significant decrease in the protein expression of cell proliferation markers Ki67 and PCNA with concomitant increase in active caspase-3 positive staining as compared to control (Figure 5). These results thus confirm the *in vitro* growth inhibitory and apoptosis inducing potential of delphinidin to *in vivo*. In addition, we found that there was a significant decrease in markers of angiogenesis (VEGF and CD31) in tumors of delphinidin treated mice when compared with tumors of the untreated control group (Figure 6). Furthermore, the xenograft mouse model is extremely useful for preclinical studies of anticancer agents thus bolstering the significance of our findings.

**Figure 7:**
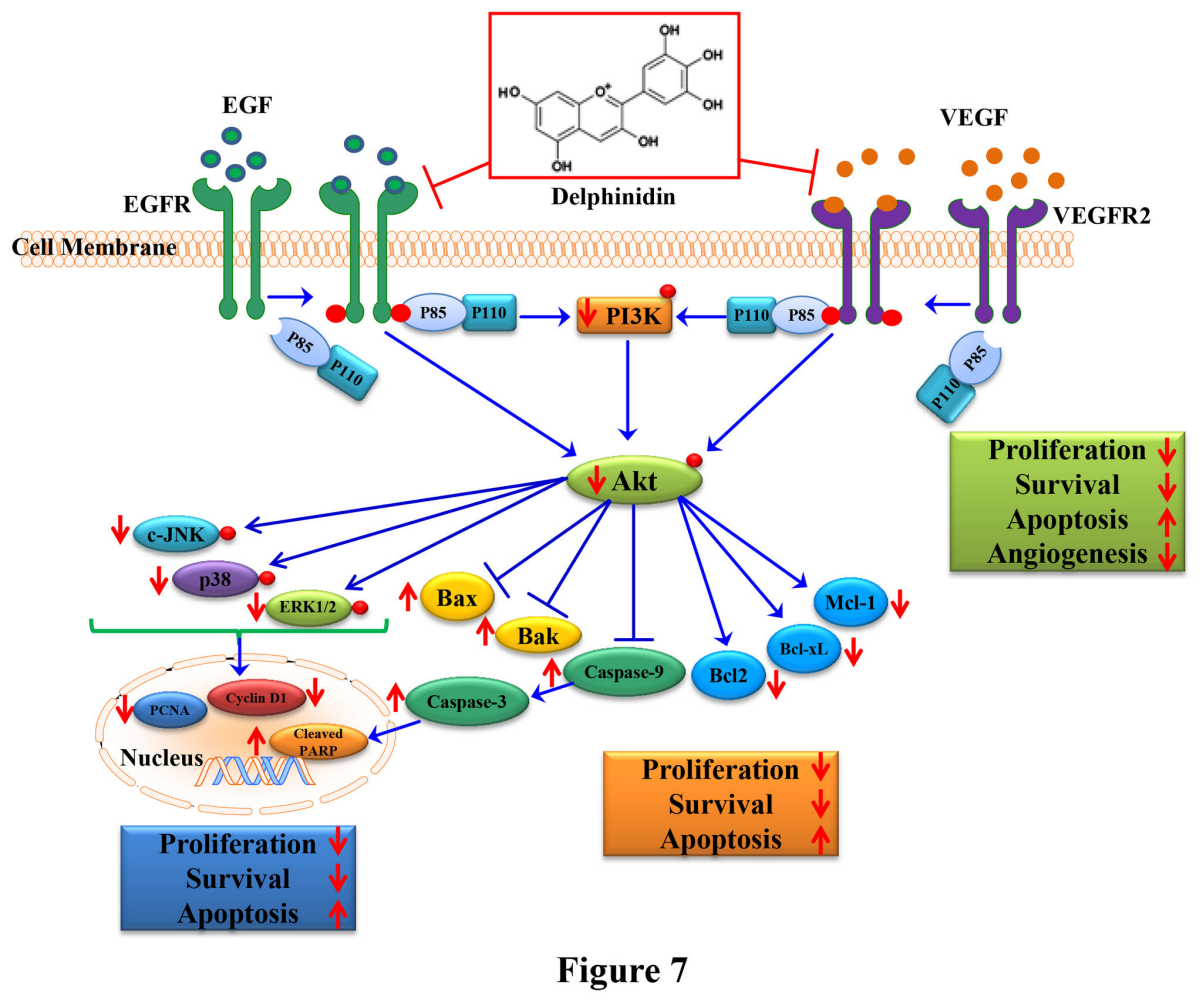
Schematic representation of the inhibitory effect of delphindin on EGFR/VEGFR2 signaling required for cell proliferation, survival, metastasis and angiogenesis.

Our study identifies delphinidin, an anthocyanidin abundant in fruits and vegetables, as an effective inhibitor of EGFR and VEGFR2 in NSCLC cells. Overall, the results from our *in vitro* and *in vivo* studies support the anti-proliferative, pro-apoptotic, anti-angiogenic and anti-tumorigenic properties of delphinidin in human NSCLC cells that overexpress EGFR/VEGFR2. The underlying mechanism by which delphinidin exhibits these activities seems to occur through inhibition of signaling pathways induced by activated EGFR and VEGFR2, such as PI3K/AKT and MAPKs (Figure 7). In summary, based on these results, we suggest that delphinidin, alone or as an adjuvant to current therapies, could be a useful agent for the management of NSCLCs that overexpress EGFR and VEGFR2.
